# Enhanced Antioxidant Activity in *Streptococcus thermophilus* by High-Level Expression of Superoxide Dismutase

**DOI:** 10.3389/fmicb.2020.579804

**Published:** 2020-11-12

**Authors:** Linghui Kong, Zhiqiang Xiong, Xin Song, Yongjun Xia, Hui Zhang, Ying Yang, Lianzhong Ai

**Affiliations:** ^1^Shanghai Engineering Research Center of Food Microbiology, School of Medical Instrument and Food Engineering, University of Shanghai for Science and Technology, Shanghai, China; ^2^Institute of Food Science, Zhejiang Academy of Agricultural Sciences, Hangzhou, China

**Keywords:** *Streptococcus thermophilus*, superoxide dismutase, native constitutive promoter, multicopy gene expression, oxidative stress

## Abstract

Superoxide dismutase (SOD) plays an essential role in eliminating oxidative damage of lactic acid bacteria. *Streptococcus thermophilus*, an important probiotic lactic acid bacterium, often inevitably suffers from various oxidative stress during dairy fermentation. In this study, to confer high-level oxidative resistance, the *sod* gene from *Lactobacillus casei* was heterologous expressed in *S. thermophilus* S-3 using our previous constructed native constitutive promoter library. The enzyme activity of SOD was significantly enhanced in engineered *S. thermophilus* by promoter #14 (2070 U/mg). Furthermore, the strategy of multi-copy *sod*-expressing cassettes was employed to improve SOD activity. The maximum activity (2750 U/mg) was obtained by the two-copy *sod* recombinant, which was 1.5-fold higher than that of one-copy recombinant. In addition, the survival rate of multi-copy *sod* recombinants was increased about 97-fold with 3.5 mmol/L H_2_O_2_ treatment. To our knowledge, this is the first report of multi-copy *sod* gene expression in *S. thermophilus*, which exerts a positive effect on coping with oxidative stress to enhance the potential of industrial application.

## Introduction

**FIGURE 1:**
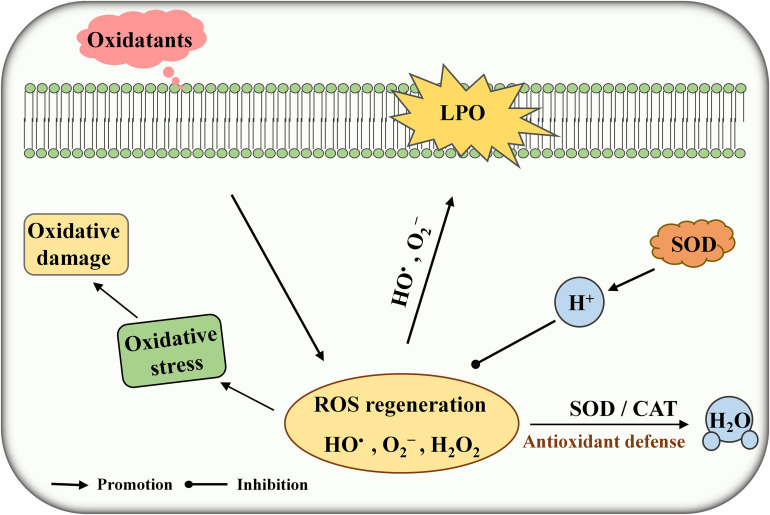
Schematic diagram of superoxide dismutase (SOD) against (reactive oxygen species) ROS toxicity. LPO, lipid peroxidation.

Under normal physiological conditions, cells can generate a massive amount of reactive oxygen species (ROS) during metabolism in the presence of oxygen. ROS including superoxide (O_2_^–^), hydroxyl radical (HO^•^), and hydrogen peroxide (H_2_O_2_) may cause lipid peroxidation, cell membrane damage, inflammation, and autoimmune diseases (Li et al., 2019). Superoxide dismutase (SOD) is one of the major antioxidant enzymes responsible for protecting the cells against the harmful effects of ROS (Figure 1). SODs are usually grouped on the basis of metal content such as CuSOD, MnSOD, and FeSOD. FeSOD and MnSOD are belonging to a homologous group and playing an important role in the living organism (Russo Krauss et al., 2015). Some lactic acid bacteria (LAB) possess cambialistic SOD, which can bind and exchange Fe or Mn in the active site and show substantial but different activity (De Vendittis et al., 2010). *Streptococcus thermophilus* AO54 and LMG 18311 contain a single MnSOD which are not sensitive to H_2_O_2_ treatment in the presence of high Mn concentration (Andrus et al., 2003; De Vendittis et al., 2012). Serata et al. (2018) found a correlation between SOD activity and Mn concentration in *Lactobacillus casei* Shirota. Thus, enhancing SOD expression is an efficient method to reduce oxidative stress in living cells. For example, heterologous expression of SOD can significantly improve the tolerance toward H_2_O_2_ stress in LAB such as *Bifidobacterium longum*, *L. casei*, and *L. rhamnosus* (An et al., 2011; Lin et al., 2016).

*S. thermophilus* is one of the homofermentative LAB commonly used in food fermentation including yogurt and cheese, which helps food safety, taste, and quality (Sorensen et al., 2016). Furthermore, it has latent beneficial effects on human health, such as promoting lactose digestion, modulating gut microbiota, alleviating intestinal disorder, and lowering blood pressure and cholesterol (Zhai et al., 2020). *S. thermophilus* is inevitably exposed to various environmental pressures during food production and beverage consumption. Accordingly, tolerance to oxidative stress is critical for the survival of *S. thermophilus*. So far, there are limited studies on SOD expression for improving antioxidant activity in *S. thermophilus*.

*S. thermophilus* S-3 was isolated from traditional dairy products by our laboratory. It can utilize galactose, which is an ideal property of LAB (Xiong et al., 2019a). Exopolysaccharide produced by S-3 effectively improves the viscosity and taste of yogurt (Xu et al., 2018). In our previous study, a set of constitutive promoters with different strengths was mined by the analysis of RNA and genome sequencing in S-3 (Kong et al., 2019; Xiong et al., 2019b). In this study, a native constitutive promoter library and multi-copy strategy were used to improve enzyme activity of SOD and the survival under H_2_O_2_ stress in S-3. Our results provide protection strategy for *S. thermophilus* or other LAB from oxidative damage.

## Materials and Methods

### Bacterial Strains and Growth Condition

Bacteria and plasmids used in this study are listed in Table 1. *Escherichia coli* Top10 was grown in Luria-Bertani (LB) medium at 37°C. *S. thermophilus* S-3 was cultured in M17 medium supplemented with 2% lactose (LM17) at 37°C. When required, erythromycin (10 μg/mL for *S. thermophilus*) and kanamycin (50 μg/mL for *E. coli*) were added.

**TABLE 1:** Strains and plasmids used in this work.

Strains	Description	Sources
*E. coli* Top10	Host for cloning	Our lab
*S. thermophilus* S-3	Wild type	Our lab
*L. casei* LC2W	The source of SOD	Our lab
**Plasmids**		
pKLH32	pIB184 derived, carrying Kan^r^ from pnCL03	Our lab
pKLH295	pKLH32 derived, for overexpression of *sod* under the #1 promoter	This study
pKLH296	pKLH32 derived, for overexpression of *sod* under the #2 promoter	This study
pKLH297	pKLH32 derived, for overexpression of *sod* under the #3 promoter	This study
pKLH298	pKLH32 derived, for overexpression of *sod* under the #4 promoter	This study
pKLH299	pKLH32 derived, for overexpression of *sod* under the #5 promoter	This study
pKLH300	pKLH32 derived, for overexpression of *sod* under the #6 promoter	This study
pKLH301	pKLH32 derived, for overexpression of *sod* under the #7 promoter	This study
pKLH302	pKLH32 derived, for overexpression of *sod* under the #8 promoter	This study
pKLH303	pKLH32 derived, for overexpression of *sod* under the #10 promoter	This study
pKLH304	pKLH32 derived, for overexpression of *sod* under the #15 promoter	This study
pKLH305	pKLH32 derived, for overexpression of *sod* under the #16 promoter	This study
pKLH306	pKLH32 derived, for overexpression of *sod* under the #17 promoter	This study
pKLH161	pKLH32 derived, for overexpression of *sod* under the #9 promoter	Kong et al., 2019
pKLH162	pKLH32 derived, for overexpression of *sod* under the #11 promoter	Kong et al., 2019
pKLH163	pKLH32 derived, for overexpression of *sod* under the #12 promoter	Kong et al., 2019
pKLH164	pKLH32 derived, for overexpression of *sod* under the #13promoter	Kong et al., 2019
pKLH165	pKLH32 derived, for overexpression of *sod* under the #14 promoter	Kong et al., 2019
pKLH167	pKLH32 derived, for overexpression of *sod* under the #18 promoter	Kong et al., 2019
pKLH168	pKLH32 derived, for overexpression of *sod* under the P_32_ promoter	Kong et al., 2019
pKLH337	Derived from pKLH167, for overexpression of *sod.*	This study
pKLH341	Derived from pKLH337, for overexpression of *sod.*	This study
pKLH344	pKLH341 digested by *Sac*I, for overexpression of *sod*.	This study

### Plasmid Construction

All primers used in this study are listed in Table 2. All molecular manipulations were carried out using standard cloning techniques (Kong et al., 2019). The DNA fragment of SOD was amplified from *L. casei* by PCR using a pair of primer SOD_LC2W-F71/SOD_LC2W-R97 and inserted into pKLH71 and pKLH81 (digestion by *Bam*HI and *Bgl*II) to obtain pKLH295 and pKLH303, respectively, by the ClonExpress MultiS one-step cloning kit (Vazyme, Nanjing, China). As the above method, the gene of *sod* was cloned into pKLH73-pKLH79 and pKLH86-pKLH88 containing different promoters by digestion of *Bam*HI and *Eco*RI (Kong et al., 2019), yielding the plasmids pKLH296-pKLH302 and pKLH304-pKLH306. Promoters name and sequence are listed in Supplementary Table 1.

**TABLE 2:** Oligonucleotides used in this study.

Primers	Sequences (5′-3′)
SOD_LC2W-F71	aaggagagtattgtaggatccATGACATTTGTTTTGCCAGATTTACC
SOD_LC2W-F73	tagggggatacagaaggatccATGACATTTGTTTTGCCAGATTTACC
SOD_LC2W-F74	ggaggcttttcctaaggatccATGACATTTGTTTTGCCAGATTTACC
SOD_LC2W-F75	taggagatccaaactggatccATGACATTTGTTTTGCCAGATTTACC
SOD_LC2W-F76	aggagaattttgcaaggatccATGACATTTGTTTTGCCAGATTTACC
SOD_LC2W-F77	aaaggggaagacattggatccATGACATTTGTTTTGCCAGATTTACC
SOD_LC2W-F78	taaggagaatacggtggatccATGACATTTGTTTTGCCAGATTTACC
SOD_LC2W-F79	tatgatatcgagatgggatccATGACATTTGTTTTGCCAGATTTACC
SOD_LC2W-F81	gaagacattattttcggatccATGACATTTGTTTTGCCAGATTTACC
SOD_LC2W-F86	aggaggaaatcactaggatccATGACATTTGTTTTGCCAGATTTACC
SOD_LC2W-F87	aaggagatagaacatggatccATGACATTTGTTTTGCCAGATTTACC
SOD_LC2W-F88	cgtgtccatgcagagggatccATGACATTTGTTTTGCCAGATTTACC
SOD_LC2W-R97	cttaagcttatcgatagatctTCAGGCGTTTGTATCGGGATG
SOD_LC2W-R	agatctcgagctctagaattcTCAGGCGTTTGTATCGGGATG
pKLH167-ske-F	gaattctagagctcgagatctatcg
pKLH167-ske-R	tcaggcgtttgtatcggg
ske1717-SOD-F	atcccgatacaaacgcctgaAAAGATATTTAAAAAGAGTGAGTACTGGG
ske1717-SOD-R	gatctcgagctctagaattcTCAGGCGTTTGTATCGGG
ske1717-SOD-R1	cattctttgactaatcactttcaggcgtttgtatcggg
ske1717-SOD-F1	cgagatctatcgataagcttaaagatatttaaaaagagtgagtactggg
pKLH337-ske-R	aagcttatcgatagatctcgagctct
pKLH337-ske-F	aagtgattagtcaaagaatggtgatgacaattg
pKLH314-SOD-F	gatacaaacgcctgagagctcAAAGATATTTAAAAAGAGTGAGTACTGGG
pKLH314-SOD-R	ttatcgatagatctcgagctcTCAGGCGTTTGTATCGGGAT
SOD-real-F	TGAACCTTACATTGACGCAACAACG
SOD-real-R	AGTTGCTCAATGGATTTGCCGG

Promoter #14 and SOD (pKLH167-ske-F/ske1717-SOD-R) were amplified from pKLH167 and ligated into the backbone of pKLH167 using a pair of primer pKLH167-ske-F/pKLH167-ske-R, yielding pKLH337. Then, promoter #14 and SOD were amplified from pKLH167 and ligated into the backbone of pKLH337, yielding pKLH341. Finally, the promoter #14 and SOD were digested by *Sac*I and inserted into plasmid pKLH341 to create pKLH344.

### RNA Preparations and Real-Time Quantitative PCR Analysis

The cells of *S. thermophilus* recombinants were collected at 24 h and total RNA was extracted with a Trizol kit (Takara). The quantification and integrity of RNA were determined by Nanodrop2000. The cDNA prepared with PrimeScriptRT reagent kit (Takara) was used as a template for RT-qPCR. RT-qPCR was performed using SYBR Premix Ex Taq (Takara). The primers of RT-qPCR are listed in Table 2. The data of each gene was analyzed using 2^–△△CT^ method with *recA* as a reference gene (Xiong et al., 2019b). Three independent biological replicates were performed for RT-qPCR experiment.

### Detection of SOD Activity in *S. thermophilus* Recombinants

The *sod* plasmids pKLH161-pKLH165, pKLH167-pKLH168, and pKLH295-pKLH306 as well as the control plasmid pKLH32 (without *sod*) were transformed into S-3 by electroporation. *S. thermophilus* transformants were harvested at 24 h. The transformants were cultivated for 24 h at 37°C and harvested by centrifugation at 8000 × *g* for 2 min at 4°C. The samples were suspended in PBS (pH 7.0) for ultrasonic pretreatment (BRANSON S-450D, United States; pulse on 4 s, pulse off 6 s, total time 40 min), and cell debris were removed by centrifugation (12000 × *g*, 2 min, 4°C). The supernatants were collected as enzyme solution. SOD activity was determined using a WST-1 SOD assay kit (Nanjing Jian Cheng Co., China) according to manufacturer recommendation. A mixture of 200 μL substrate application solution, 20 μL of enzyme working fluid, and 20 μL of enzyme solution was incubated at 37°C for 20 min, and measured using spectrophotometry at 450 nm. The concentration of total protein was determined by modified Bradford reagent (Sangon Biotech, China) using bovine serum albumin as the standard. One unit (U/mg) of SOD activity was defined as the amount of enzyme per mg of total protein corresponding to the SOD inhibition rate reaching 50% in the reaction system (Kong et al., 2019).

### Growth of *S. thermophilus* Under Oxidative Stress Conditions

The multi-copy *sod* recombinants were cultured in LM17 medium containing erythromycin at 37°C for 12 h. Then the seeds were inoculated (3% v/v) into fresh LM17 medium broth supplemented with H_2_O_2_ (1 and 2.5 mmol/L) to determine their growth under aerobic conditions, respectively. Furthermore, colony-forming units per milliliter (CFU/mL) was obtained by taking 10 μL stationary phase cultures (10-fold serial dilutions) on LM17 plate containing H_2_O_2_ (3.5 mmol/L) at 37°C for 48 h (anaerobic conditions), respectively.

## Results and Discussion

### Heterologous Expression of SOD Using Native Constitutive Promoter Library in *S. thermophilus*

The food-grade *S. thermophilus* is a facultative anaerobic bacterium and often exposed to oxygen stress during the fermentation of yogurt or meat products. SOD has demonstrated to protect LAB from oxidative stress (Chang and Hassan, 1997; Zhai et al., 2020). Expression of the *sod* from *L. casei* (*sod*-lc) has significantly increased the activity of SOD and superoxide radical scavenging activity in *E. coli* (Liu et al., 2011). Additionally, the functional gene from *L. casei* could be more suitable for heterologous expression in *S. thermophilus* because *L. casei* and *S. thermophilus* are belonging to the group of LAB. Thus, to enhance SOD activity in S-3, we selected *sod*-lc for heterologous expression using our previous constructed native constitutive promoter library (Kong et al., 2019). The strength of 18 promoters including 7 strong promoters (#6, #9, #11, #12, #13, #14, and #18) and 11 weak promoters (#1–#5, #10, and #15–#17) were detected in Supplementary Table 1.

**FIGURE 2:**
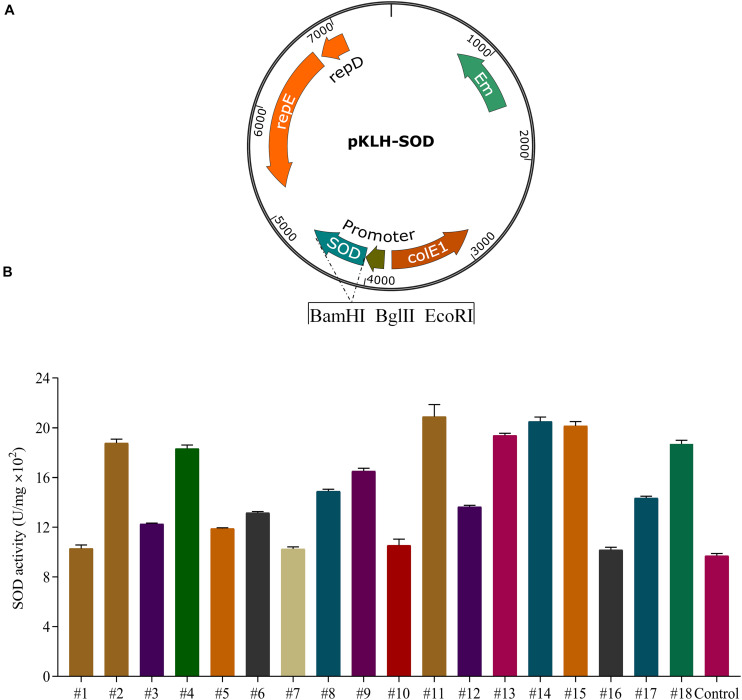
Expression of superoxide dismutase (SOD) in *S. thermophilus* S-3. Schematic representation of recombinant expression vectors containing *sod* gene from *L. casei* LC2W **(A)**. Application of a native constitutive promoter library from *S. thermophilus* for the expression of SOD **(B)**. SOD expression by the selected 18 promoters in S-3, using wild type with empty plasmid as control. The error bars indicate the standard deviations from three independent replicates.

For the recombinants of *S. thermophilus* (Figure 2A), the activities of SOD were increased 1.1–2.2 folds compared with the control strain S-3/pKLH32 (empty plasmid) under constitutive promoters with different strength (Figure 2B). Strong promoters (#11 and #14) markedly increased 2.2-fold of SOD activity compared with that of the control. The highest enzyme activity of SOD was 2070 U/mg. Interestingly, overexpression of *sod* by weak promoters (#2, #4, and #15) also significantly enhanced 1.95, 1.93, and 2.12 folds of enzymatic activity. It might be attributed that the ribosomal binding sites of these promoters are more suitable for SOD expression. These results showed that 8 recombinant strains using promoters #2, #4, #9, #11, #13, #14, #15, and #18 were superior to the currently reported highest SOD activity (1500 U/mg) of *S. thermophilus* CRL807 (Del Carmen et al., 2014). Our previous study found a higher activity of SOD by promoter #14 in comparison with promoter #11 in *S. thermophilus* and *L. casei* (Kong et al., 2019). Therefore, we selected promoter #14 for further fine-tuning *sod* expression.

### Effect of Multi-Copy *sod-*Expression Cassettes in *S. thermophilus*

**FIGURE 3:**
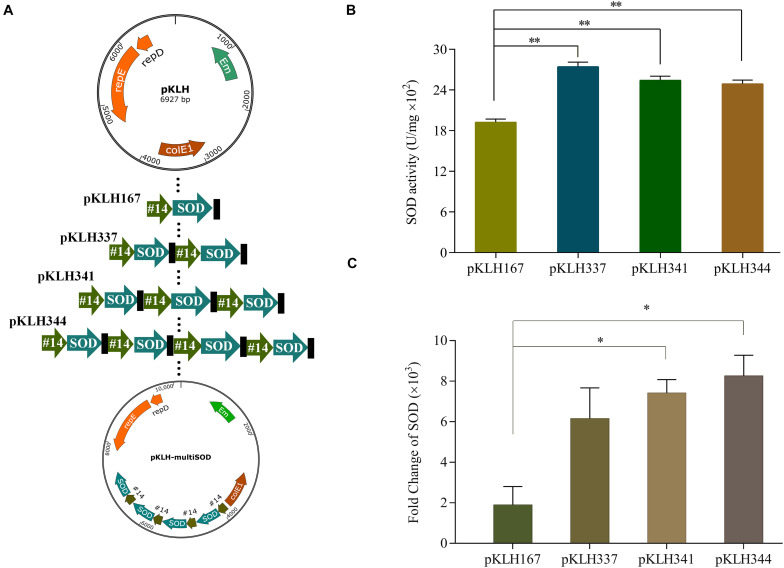
Expression of multi-copy *sod* in *S. thermophilus* S-3. Construction of vectors with increasing copy of the *sod* expression cassette **(A)**. Analysis of the enzyme activity of superoxide dismutase (SOD) in recombinants **(B)**. Analysis of the transcriptional level by RT-qPCR in recombinants **(C)**. The error bars show the standard deviations from three independent replicates. *P < 0.05, **P < 0.01.

Multi-copy expression cassette is an important strategy to significantly enhance the expression of recombinant proteins (Zhu et al., 2009). Wang et al. (2014) applied this strategy to achieve the maximum yield at the three copies of *BmK1* gene, which improved 2.09-fold compared with the control. However, an optimal gene copy number should be considered because of metabolic burden by protein overexpression (Rawsthorne et al., 2006). In some cases, it results in reducing the expression of protein by further increasing gene copy number. Here, multi-copy expression cassette was used to further enhance the expression of SOD. One to four copies of *sod*-lc gene expression were constructed under promoter #14 (Figure 3A), which one to four copy number recombinants named as S-3/pKLH167, S-3/pKLH337, S-3/pKLH341, and S-3/pKLH344. The maximum activity of SOD was obtained by S-3/pKLH337 (2750 U/mg), which improved 1.45-fold compared with that of S-3/pKLH167 (Figure 3B). SOD activity of S-3/pKLH337 was ∼1.8-fold higher than that (1500 U/mg) of *S. thermophilus* CRL807 (Del Carmen et al., 2014). SOD activities of S-3/pKLH341 and S-3/pKLH344 were also significantly increased 1.34 and 1.32 folds, respectively. Due to metabolic burden caused by protein overexpression, SOD expression does not increase indefinitely with an increase in copy number. Shu et al. (2016) had a similar result that two copies of expression cassette achieved the highest activity of serine protease, not the four copies. It suggested that the multi-copy strategy is efficient for the expression of SOD and other functional proteins.

Due to the highest expression of SOD in two copies recombinant, the relationship between gene copy number and transcription change were evaluated by RT-qPCR. The expression of *sod* in all multi-copy recombinants were remarkably increased >2000-fold at the transcriptional level compared with the empty plasmid strain (Figure 3C). Gene expression of *sod* were increased 3.9, 5.2, and 5.9-fold in multi-copy recombinants S-3/pKLH337, S-3/pKLH341, and S-3/pKLH344 compared to one copy S-3/pKLH167. It indicated that gene copy number has strongly correlated with the level of *sod* expression, not SOD activity. Our result was similar to the report by Shu et al. (2016) that higher gene copy number increased gene expression but not protein expression level.

Some bacteria possess cambialistic SOD capable of function with Fe or Mn in the active site (De Vendittis et al., 2010). The metal cofactor Mn and Fe are critical for SOD activity (Russo Krauss et al., 2015). To assess the effect of metal ions on SOD activity, we added 1 mmol/L Fe or Mn in the culture medium of *S. thermophilus* recombinants. The addition of Mn increased SOD activity of S-3/pKLH337 by 1.7-fold, up to 4723 U/mg (Table 3). In particular, SOD activity of S-3/pKLH337 was also 4.7-fold higher than that of control strain (S-3/pKLH32). However, addition of Fe resulted in 31% and 22% decrease of SOD activity in S-3/pKLH32 and S-3/pKLH337, respectively. It is consistent with the result by Chang and Hassan (1997) that Fe inhibits enzymatic activity of SOD. Russo Krauss et al. (2015) showed that Mn-bound form of SOD from *S. thermophilus* has a significantly higher enzymatic catalysis with respect to the Fe form. Our results suggested that SOD in *S. thermophilus* is more preferred to Mn, which significantly improved enzyme activity.

**TABLE 3:** Effects of Mn or Fe supplement on SOD activity in engineered *S. thermophilus* S-3.

Strain	Metal supplement	SOD (U/mg) ± SD
S-3/pKLH32	None	1013.1 ± 36.9
	1 mmol/L Mn	1530.1 ± 43.2
	1 mmol/L Fe	702.6 ± 23.8
S-3/pKLH337	None	2651.4 ± 85.8
	1 mmol/L Mn	4723.0 ± 99.5
	1 mmol/L Fe	2065.8 ± 64.6

### Impact of SOD on Oxidative Stress Resistance in *S. thermophilus*

**FIGURE 4:**
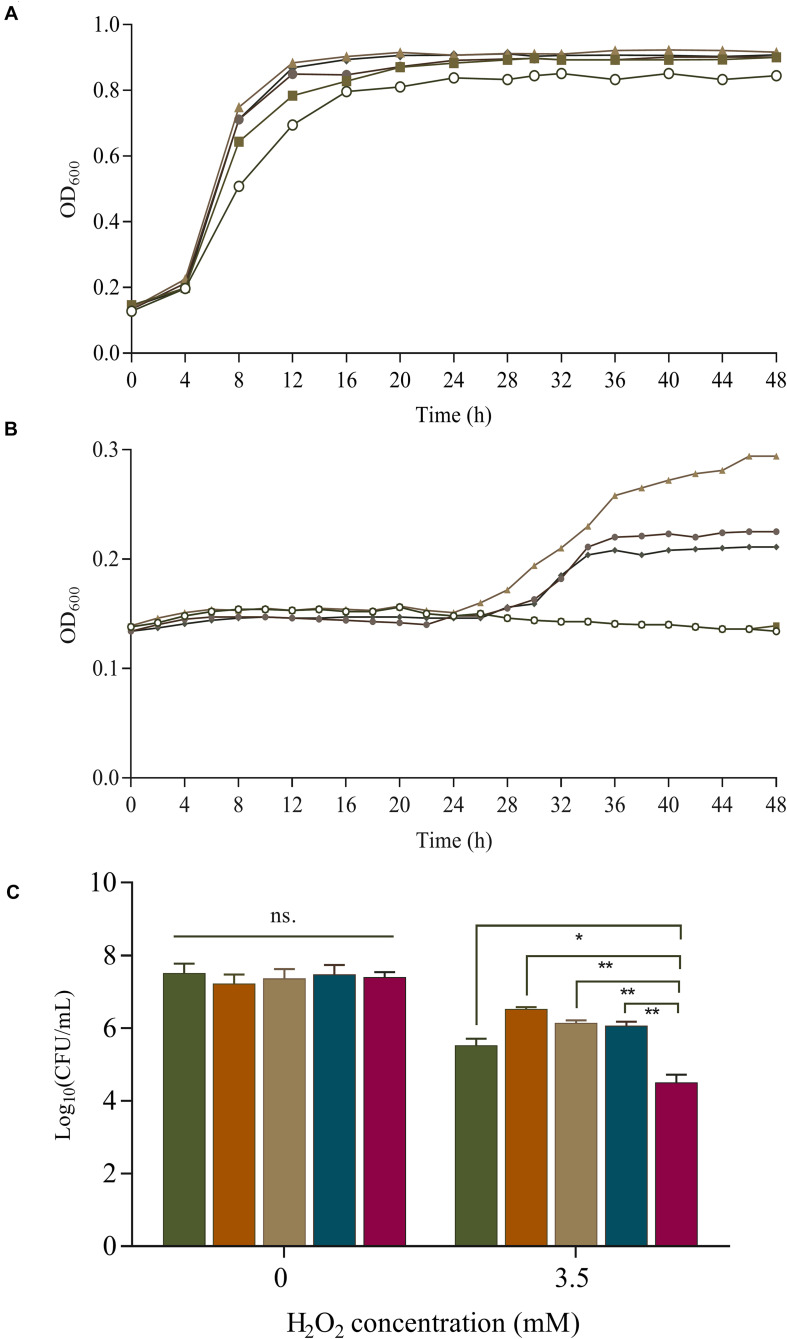
Survival of *S. thermophilus* strains under oxidative stress. Effect of superoxide dismutase (SOD) expression on cell growth of recombinant strains (one to four SOD copy numbers) under 1 mmol/L **(A)** and 2.5 mmol/L H_2_O_2_ treatment **(B)**. Symbols: 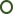, Control; 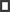, S-3/pKLH167; 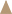, S-3/pKLH337; 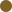, pKLH341; and 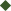, pKLH344. Viable cells under 3.5 mmol/L H_2_O_2_ treatment under anaerobic conditions **(C)**. 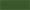, pKLH167; 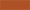, pKLH337; 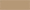, pKLH341; 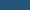, pKLH344; and 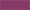, Control. *P < 0.05, **P < 0.01.

Overexpression of SOD increases the survival rate of *B. longum* NCC2705 under 2.5 mmol/L H_2_O_2_ stress during long-term cold storage (Zuo et al., 2014). Bruno-Barcena et al. (2004) found that the expression of *sod* in *L. gasseri* and *L. acidophilus* could protect against H_2_O_2_ (1.4 mmol/L) and increase their growth rate by 30% and 100%, respectively. The effect of SOD on H_2_O_2_ tolerance were investigated in our *sod*-overexpressing recombinants. Under aerobic condition, SOD overexpression boosted the growth of S-3/pKLH167, S-3/pKLH337, S-3/pKLH341, and S-3/pKLH344 with 1 mmol/L H_2_O_2_ treatment compared with the control strain S-3/pKLH32 (Figure 4A). Multi-copy *sod* recombinants S-3/pKLH337, S-3/pKLH341, and S-3/pKLH344 were able to withstand higher concentration (2.5 mmol/L) of H_2_O_2_ (Figure 4B). Our recombinants showed higher tolerance to H_2_O_2_ in comparison with other LAB such as *L. plantarum* and *Pediococcus pentosaceus* (Lin et al., 2018).

Oxidative stress resistance was further investigated by the survival rate under anaerobic condition. Significant difference of the survival was observed between the recombinants and the control. The survival rate of S-3/pKLH167, S-3/pKLH337, S-3/pKLH341, and S-3/pKLH344 were 73, 90, 83, and 81%, respectively, which were higher than that of wild-type (55%) under 3.5 mmol/L H_2_O_2_ (Figure 4C). The viable cells of S-3/pKLH337 was 9.5 and 97 folds higher than that of S-3/pKLH167 and S-3/pKLH32, respectively, which has the strongest antioxidant capacity among all strains. It suggested that multi-copy *sod* expression remarkably increases the viability of *S. thermophilus* under H_2_O_2_ stress. This is the first report on combining constitutive promoter library and multi-copy strategy to enhance antioxidative ability in *S. thermophilus* and other LAB.

For dairy fermentation, microorganisms lacking SOD is more susceptible to oxidative stress. Andrus et al. (2003) found that SOD is essential for the growth of *S. thermophilus* AO54 under oxidative stress. *S. thermophilus* with *sod* deficiency cannot grow aerobically. Lin et al. (2016) reported that adequacy activity of SOD might enhance resistance to H_2_O_2_ (2 mmol/L) and protect *lactobacillus* from oxidative stress. Bruno-Barcena et al. (2004) found that overexpression of SOD increases the growth rate from 40% to 70% compared with the wild type in *L. gasseri* under 1.4 mmol/L H_2_O_2_ stress. Compared with these strains, our multi-copy strains showed stronger tolerance to H_2_O_2_ (anaerobic 3.5 mmol/L and aerobic 2.5 mmol/L), indicating marked improvement of antioxidant capacity. Our result also suggested that SOD expression in LAB has great application potential in food industry.

## Conclusion

*S. thermophilus* often suffers from inevitable oxidative stress during food production. To deal with oxidative stress, a heterologous *sod* was successfully expressed in *S. thermophilus* using a native constitutive promoter library. Moreover, the multi-copy *sod* strains were constructed and significantly improved the activity of SOD (2750 U/mg), which is the highest enzyme activity reported in *S. thermophilus*. Interestingly, compared with the control, the number of viable cells was markedly enhanced 97-fold by multi-copy *sod* recombinants. Therefore, it indicated that the multi-copy strategy is a reliable and efficient method for improving oxidative stress resistance in *S. thermophilus*.

## Data Availability Statement

The original contributions presented in the study are included in the article/Supplementary Material, further inquiries can be directed to the corresponding author.

## Author Contributions

LA designed the experiments. LK and ZX performed the experiments and wrote the manuscript. XS, YX, HZ, and YY analyzed the results. All the authors read and approved the final manuscript.

## Conflict of Interest

The authors declare that the research was conducted in the absence of any commercial or financial relationships that could be construed as a potential conflict of interest.
