# Supplementary material for: Enhanced Antioxidant Activity in Streptococcus thermophilus by High-Level Expression of Superoxide Dismutase
Source: Front Microbiol. 2020 Nov 12;11:579804. doi: 10.3389/fmicb.2020.579804 (PMC7688587; doi:10.3389/fmicb.2020.579804)
Supplement: Supplementary file 1 [file Table_1.DOCX]

***Supplementary Material***

**Table S1** Sequences of promoters used in this study

| Name | Length of promoter | Promoter | Sequence 5’→3’ |
| --- | --- | --- | --- |
| orf0055 | 208 bp | #1 | AGGGAATCTCCTGTCTCTTTTCTATTTACCATTATACAATATTTTGAATTCCTTTTCATTTGCCTTATTTTTTCTGAGATTTTCAAAAATAATTGAAATATTAAAAAATAGATAATCGCAAGAAAATTCTGGTTTTAATTTGCTTAACCCTTGATATTATGGTAATATAAATGTGTAAAAAATTTTAACTCATAAGGAGAGTATTGTA |
| orf0136 | 239 bp | #2 | GAGCAAATATTTCTTTCGAAGCTTTTTGGAGAAACTGTGGACAACTCTTTAGAAAAAACGTTGTGTTCTTCGTTTTTTTAGTTTTTTACCACATAATTTTCAAAGGGTGCATATATCTCCATTAACATAAGATATGAAATCGGTTATATCAAAAGCTGTAGCTTTTTTATTTTCAAGTTCAGATATTGGCAGTCTGAAAAATTAAATGATAGAATGGCAGTATATAGGGGGATACAGAA |
| orf0207 | 237 bp | #3 | CATACGACTTCTTTTTGTCTTTTGGAGTGTAGTCATGGTTTGGATAGAATGAAAAGATTATAAAAGTAGTAGAAAAAGTATTATTGAAAGAAATATTTGTGATTACTATAAAGAAAAACTGAAAACTATATAAAATACTTGCAATTCTATGACATTTTGATAGAATAGTAGAGTAAAGTTAGACTGTATTGCCTACTGTCTATCTATAAAATATATTTTATTGGAGGCTTTTCCTAA |
| orf0424 | 277 bp | #4 | GGCTTCTTGCCTCCTTTCTCAAATTTATCTCATTGTAAAGCCTTTCACATAAAACTTTGGGCCTACAAAACATTCTCCCCCTATGATACCAAAATCTATCAAAAAACAGAAAATTATAATACAGATTTCCAGTTTTCAAAACTGTATTATAAAAGAATGTCTTTAAAAACGGAGTTTCAAAGTTATGTAAGCGGTTACTGTGTGATATAATACTTCTAGAGAATGTAACAGATGATTTTGTTACATTCTCTAGAAAGTATTATAGGAGATCCAAACT |
| orf0495 | 248 bp | #5 | ATTTAATAATCTCCTAATTTATTTATTAGTAATATAGTAAACGTTTTACCTAGAAAAATCAACGATTTATAATAAGAAAACAAAAAAATGCTAAACGTTTGACATATGACAGAAAGATGTTAAAATTAATATCGTAAAGAAAAGTGAAACGTTTTCAAAAACAAATTTTGTTAAAGATGTTAAAATTGATATCGTAAAGAAAAGCGAAACGTTTTCAAAAACAAATTTTATTAAGGAGAATTTTGCAA |
| orf0688 | 657 bp | #6 | AAAACCTGTTTTATATCCATTAATTTCTGAGCATAAACCAACGACCTCATCCTTATTAATTTTGGCCAATTCCTAACGCATTTCAAGCAAATTCTTGTCTCTAAATACTCGTTCAAAGCCCTCAAGCTATTTACTAAATTTGTCTAATTTAGTGTAATCCTGACAATTCAATGTTTCTCTAAAATCCATAATTATCTCCTTTTTTGAATTTTAAACTCCTACTGAAAATAACACAGTATCCTGTGTATCATTTAGACTTCTTTAATTGGCTAAAAATCTTATGGTTATACCACCATAATCAAAGGTTTTATTGATGAATTATTCAATTGTCAGAATGGTCTGATAATAATAACCATAATGAAGAGTATTTACAAAACTGTTCAAAAAATAACAAGAATCATATCAGAAATATACTTGCAAATATATGCAATCGATTGCAAAACAATTAACAGATTTCATTCTATTTTTTTATAAGCGAGATCATTCCTTTGACTTATCATTTTTATTGACCTATACTATTCGCAAAGATATATACTAACATTAATCTTATGATTGTTAAGATTAATATAAAAAATATTTTAGCTAAAATATAGCTCTTTAGATTGTTTGATTTTCCAAACAATCTAACTTGCCACAATTTAGAAAGGGGAAGACATT |
| orf0847 | 147 bp | #7 | GATTTTAGACTCTAATTCAGAGTCTTTTTTATAAAGTTCGGAAATAGGACTAATAATTTTTTAATAAAAGTCAGAAAATTGAAAAATAGCTGACGATTTACAGAAAAACAGTTTACTTTTGTTTTAAAAGTGATATCATAAAAGGGTACTACATAAAGTAAGGAGAATACGGT |
| orf0852 | 197 bp | #8 | ATAAGAGTCTAAGTCAAATAGACTTGGTCTTTTTGTCTAGGTACATAATAATCTATAAAGGTTTTCAAAATTATCTTGTCATGCCTTAAGCTTTTTGCTAAAATATTTCTATGAAAACATTATATGACGTTCAACAGTTGTTGAAGCAGTTTGGGGTAGTAGTTTACTTGGGTAAAAGGCTCTATGATATCGAGATG |
| orf0918 | 531 bp | #9 | ATAAACACCTCATCGGTTTTCTTAAGTCATTCAATTAGTAAAGCCAAAAACTTTACATATCAAACATATTATACCAAAAAATAGGTCAAGGACCAAGTTTCGAGTATGTCTTTGTGGCTGATATATAAGCTTTCTTTACATTTTTATTAATGAGCAATCACCAACCTTCCTTAGACTAAATATTTCTGACTTTTACAACATCTACAGTAGAATAACTAGTTCTAGTACATTTAGTTACAAAATATGAAATTAACCATCGTTTTAACTTGTTTCGTACTTAACTTTACCACCTCTAACAACTCTCGTTACCCCAGCAAACAATGGTAACAAGCTAGTGAAGGCAGTACTGATCCTAATGCCGATTTTTTTCTATATCTATATCTATATCGTAAAATTAGTTAACTATATCATAAAAACCGAACATTTCGCAAAAATATTCGTATTTTTTGACTTTAAACTCATCTTATGCTAAACTAAAAAATATGAACATGTTAGAAAATCTAACAAGCAAATCCATTATAGGAGGCTCTT |
| orf0955 | 152 bp | #10 | GAAGGGCTCCTTTCAACATTATTTTTTGTCAAATAATAATTTAGGTTTCTAGAAAAAATTAAAGAAATCTATTCAAATAGTATTATATCACAAGTTTTTAAGCATATTAAAAGCAGAACCAAGGGTTCTGCTTTCTTGAAGACATTATTTTC |
| orf0964 | 281 bp | #11 | CGTCGTAGATGTGCAAGGTGAAAAAAAGAAAAACCTCACTGTTGCAGAGCTTATGGCCCTCTTCCACAAAAATAGTGGACAAGTTCTCAATGACGATGCCTTGGTACTCGGTTAATATACTAGAAAAAAACTATCCAAAGTTTAGATTTTGGATAGTTTTTTCTTGACTATTTTTGACTAAGTGCTAAAATGAAAATATAGATTAGCAGTTGTCTATCGTGAGTGCTAAAACAACATTTTAAACGTTGCTAATCTAAATAACATTTTTGGAGGTAATTGGT |
| orf1135 | 168 bp | #12 | CCAATCCCCCTCCTTTTTTCCCTATTTTACAAAAAAAGTGACAAAATTGTAAAGATAAGTAAAAGACTTACAGTTTCTCGTATAAATCTAGGTGCTAAGTCCATAAATTTGTGATACAATGATTGCATAAGAAAGAATCCATATGTGATGCAAAAAGAAAGGGTAATA |
| orf1396 | 210 bp | #13 | AAAAGGTTTAAATATTTACTTTTGGTTGGTAACTTAAGTTAGCAACCATTTTTTATTACCTAGACAATCGTTTTTCAGAAAATCGACAATCGTTTTTCAGAAAATCCAGAGCACTAAATTCTTGACACGTGCAACCGTTTGCATTATAATCGCTAGTGTAAAAAATAATTCGAGGTAAACTTTATGAACATACGAGCAAGCGGTGTTTTA |
| orf1717 | 232 bp | #14 | AAAGATATTTAAAAAGAGTGAGTACTGGGAAAAGGTATTCACTCTTTTTAATAGTGTCCTCACCTTGAAAAAAGTCGCTTTTATTGATAGAATGGTAAGAGAATAAGGCTGCTTTGAAAAGTGAGCTGTAACTTAATCACTGAACAACCCCTAAACCATAAAAAGAAATGATTTACTTCTCTTTTTATGGTAAGTCATAATAAGAATTTTAATCTTAGTTTAAGGAGAAGAC |
| orf1746 | 186 bp | #15 | TTAGAAGTTTTTAAGTTAAGGTGCTTATGAAAGTAAGTGCCTTTTTCTATTGCATTTTCCTGAAAAAAATTATATAATGGATGTGTTAAAAGGTTAAATGAAAGCCCTCAAGTTAATCTTTTCACAATAGGGAGAGCTCGCTCTCAAAATTATATTTATTTGATTTTCATAAGGAGGAAATCACTA |
| orf1768 | 128 bp | #16 | AAAACCTTGTCCGAAACGACAATAAACTATGAGAGAAAAGATAAGAGGCAAGCCGAAGGTTTGCAAAAAGAAATATCAGAAAAGAAGAAAAAAGCTTAGAAAGAAAAAAGCTTAAGGAGATAGAACAT |
| orf1769 | 147 bp | #17 | AGATATTAAAGAAGAAGAAGTAAAAGAAGNGAAGCTACCGAGACAACTGAAGAAGTTGTAGAAGAAACAAAGGAAACTTCTGAGCTTGAAGAAGCTCAAGCGCGTGCGGAGGAGTTTGAAAATAAATACCTTCGTGTCCATGCAGAG |
| orf1770 | 236 bp | #18 | TATTAGAACACCCCTAAGGGTGTTTTTTAATGTTTCTAGGTCTCTAGATGTATTTTTAGCACTCTTTCAAAAAGAGTGCTAATTTTTTTGTTTTTTTTTGGGGTTGACATGTTTAAGTTTAGGTGTATAATAGAATCATAAGTTAGCAGTCGTTGTACGAGAGTGCTAATAAATAGAAATGAGGTGACATCGTGATTACGCAAAGGCAAAACGCTATTTTGAATTTGATAGTTGAG |
